# Adherence to international guidelines for the management of *Helicobacter pylori* infection among gastroenterologists and gastroenterology fellows in Italy: A Survey of the Italian Federation of Digestive Diseases ‐ FISMAD

**DOI:** 10.1111/hel.12862

**Published:** 2021-11-11

**Authors:** Rocco Maurizio Zagari, Marco Romano, Leonardo Frazzoni, Giovanni Marasco, Elton Dajti, Paolo Giorgio Arcidiacono, Alessandro Armuzzi, Federico Biagi, Renato Cannizzaro, Giulia Martina Cavestro, Carolina Ciacci, Fabio Monica, Sergio Peralta, Franco Radaelli, Franco Bazzoli

**Affiliations:** ^1^ Gastroenterology Unit IRCCS Azienda Ospedaliero‐Universitaria, S. Orsola Hospital Bologna Italy; ^2^ Department of Medical and Surgical Sciences University of Bologna Italy; ^3^ Division of Hepatogastroenterology Department of Precision Medicine University of Campania "Luigi Vanvitelli" Naples Italy; ^4^ Pancreatobiliary Endoscopy and Endosonography Division IRCCS San Raffaele Scientific Institute Milan Italy; ^5^ Department of Medical and Surgical Sciences IRCCS A. Gemelli University Hospital Rome Italy; ^6^ Gastroenterology Unit IRCCS Istituti Clinici Scientifici Maugeri University of Pavia Italy; ^7^ Centro Riferimento Oncologico IRCCS Istituto Nazionale Tumori Aviano Italy; ^8^ Gastroenterology and Gastrointestinal Endoscopy Unit IRCCS San Raffaele Scientific Institute Milan Italy; ^9^ Gastrointestinal Unit Department of Medicine, Surgery and Dentistry University of Salerno Italy; ^10^ Gastroenterology and Digestive Endoscopy ‘Cattinara’ Academic Hospital Trieste Italy; ^11^ Gastroenterology and Hepatology Unit University of Palermo Italy; ^12^ Gastroenterology Unit Valduce Hospital Como Italy

**Keywords:** diagnosis, gastroenterologists, gastroenterology fellows, guidelines, *Helicobacter pylori*, treatment

## Abstract

**Background:**

Information on the management of *Helicobacter (H*.*)* *pylori* infection by gastroenterologists and gastroenterology fellows are scarce. We aimed to assess practice of gastroenterologists and gastroenterology fellows and their adherence to guidelines for diagnosis and treatment of *H. pylori* infection in Italy.

**Materials and Methods:**

All gastroenterologists and gastroenterology fellows attending the National Congress of Digestive Diseases ‐ FISMAD were invited to fill‐in an on‐line questionnaire. The questionnaire included questions on the diagnosis and treatment of *H. pylori* infection.

**Results:**

A total of 279 gastroenterologists and 61 gastroenterology fellows participated to the study. The ^13^C‐urea breath test was the most preferred method among gastroenterologists and fellows for the diagnosis of *H. pylori* infection (40.4% and 57.6%, respectively) and the confirmation of eradication (61.3% and 70%, respectively). Sequential therapy was the most preferred first‐line treatment of *H. pylori* for both gastroenterologists and gastroenterology fellows (31.8% and 44%, respectively), followed by bismuth quadruple therapy (31% and 27.6%, respectively) and clarithromycin triple therapy (26.8% and 22.4%, respectively). Only 30% of gastroenterologists and 38.5% of fellows used the clarithromycin triple therapy for the recommended duration of 14 days. Bismuth quadruple therapy was the most preferred second‐line therapy for both gastroenterologists and fellows. The majority of gastroenterologists and fellows would prefer an empirical therapy at third line (72.6% and 62.5%, respectively) and a susceptibility‐guided therapy at fourth line (46.7% and 71.4%, respectively).

**Conclusions:**

Practices of gastroenterologists and gastroenterology fellows are in line with guidelines’ recommendations, apart for the first‐line treatment of *H. pylori* infection. Targeted educational interventions to improve adherence to guidelines are needed.

## 1 INTRODUCTION

Although the prevalence of *Helicobacter (H*.*)* *pylori* infection has been decreasing over the last decades, this bacterium still infects more than half of the world's population.^1^ *H. pylori* infection causes chronic gastritis, peptic ulcer and gastric malignancies, and it is also an organic cause of dyspepsia and extra‐gastric diseases.^2^, ^3^, ^4^ Thus, all patients testing positive for *H. pylori* should be offered an eradication therapy.^5^

The management of *H. pylori* infection still represents an issue in clinical practice. The use of culture or molecular test to assess antibiotic susceptibility of *H. pylori*, the treatment to prescribe, and the test to confirm eradication are still debated. In particular, the eradication of *H. pylori* is becoming more difficult due to the increasing prevalence of antibiotic resistance,^6^, ^7^, ^8^ and a number of antimicrobial regimens are now recommended.

Recent international guidelines by three separate authoritative groups from Europe, America and Canada provided evidence‐based recommendations to help physicians in the diagnosis and treatment of *H. pylori* infection,^9^, ^10^, ^11^ and a recent review reconciling guidelines showed a substantial agreement among guidelines’ recommendations.^12^ Currently, the ^13^C‐urea breath test (UBT) is considered the best method for both the diagnosis of *H. pylori* and the confirmation of eradication; testing for eradication should be performed at least 1 month after the end of therapy.^9^ As for the treatment, a 14‐day clarithromycin triple therapy is suggested only in patients who are from regions with a low prevalence (<15%) of clarithromycin resistance, whereas bismuth and non‐bismuth quadruple therapies are mandatory in settings of high (15%) or unknown clarithromycin resistance.^9^, ^10^, ^11^ Since few years, the new formulation of single‐capsule bismuth quadruple therapy is available in many countries, including Italy.^13^

Gastroenterologists play an important role in the management of *H. pylori* infection both in treating patients and in the guidance of practitioners. However, information on the practice of gastroenterologists in the diagnosis and treatment of *H. pylori* infection and their adherence to guideline recommendations is scarce. A recent study reported that treatment of *H. pylori* infection by European gastroenterologists is discrepant with current recommendation.^14^ Similarly, a survey carried out in China showed among clinicians, of whom 85% were gastroenterologists, a gap between real‐world practices and guidelines for the management of *H. pylori* infection.^15^ In addition, there is consistent evidence that compliance of also primary care physicians with *H. pylori* guidelines is low.^16^, ^17^, ^18^ It has been suggested that the poor practice of primary care physicians may be a further, albeit indirect, evidence of the suboptimal management of *H. pylori* infection by gastroenterologists.^19^

Further information on the adherence of gastroenterologists to guidelines recommendations are needed in order to optimize the management of *H. pylori* infection in clinical practice. In addition, such information could inform scientific societies on the need for targeted educational interventions, that may be effective in increasing knowledge and compliance with *H. pylori* guidelines.^20^

The aim of this study was to assess practice patterns of gastroenterologists and gastroenterology fellows and their adherence to international guidelines for the diagnosis and treatment of *H. pylori* infection in Italy.

## 2 MATERIAL AND METHODS

This is a survey conducted among gastroenterologists and gastroenterology fellows attending the 23rd National Congress of the Italian Federation of Digestive Diseases (FISMAD), that was held in Bologna, Italy, from 29th March to 1st April 2017. The FISMAD is the Federation of the three scientific societies of digestive diseases: the Italian Society of Digestive Diseases (SIGE), the Italian Association of Hospital Gastroenterologists (AIGO), and the Italian Society of Digestive Endoscopy (SIED). All gastroenterologists and gastroenterology fellows attending the congress were invited to fill‐in an on‐line questionnaire through a link uploaded in the FISMAD website (www.FISMAD.it) using dedicated computers allocated in the registration area. Responses were collected electronically during the 4 days of the Congress. Subjects not willing to participate to the study were asked to fill‐in only the first section of the questionnaire, including demographic and professional characteristics of participants. There were no incentives for the participation in the study. This study was an initiative of the Scientific Committee of FISMAD and was conducted after approval by the Governing Council of the Federation itself. Written informed consent to anonymous use of data provided in the questionnaire was individually obtained from all participating physicians.

### 2.1 Questionnaire

The questionnaire was developed according to the available international guideline recommendations on the management of *H. pylori* infection.^9^, ^10^, ^11^

The questionnaire had three sections, including a total of 16 multiple‐choice questions. The first section contained five questions regarding demographic and professional characteristics of the participants. The second section included four questions on the diagnosis of *H. pylori* infection, such as the preferred test for the initial and post‐treatment diagnosis, the interval between the end of therapy and the test for confirmation of eradication, and the availability of antimicrobial susceptibility testing, such as culture or molecular tests. The third section contained seven questions regarding the treatment of *H. pylori*, including the proportion of patients treated with a first‐line therapy, the local prevalence of clarithromycin resistance, the previous use of key antibiotics, the preferred first‐, second‐, and third‐line therapy and the management of patients after failure of three lines of treatment. The questionnaire is presented as Appendix S1.

### 2.2 Statistical analysis

We performed descriptive analyses using percentages for categorical variables. We calculated statistical differences between percentages using the Chi‐square test or Fisher's test when appropriate. A *p* value < .05 was considered statistically significant. Statistical analysis was performed using STATA version 16 (Stata Corp, College Station, Texas, USA).

## 3 RESULTS

### 3.1 Study sample

A total of 534 gastroenterologists and 140 gastroenterology fellows were eligible for the study. Of these, 279 (52.2%) gastroenterologists and 61 (43.6%) fellows completed the questionnaire. Not all participants answered to all the questions, thus the number of responses for each question varied accordingly. The majority of gastroenterologists (62.3%) practiced in community hospitals, whereas 25.2% worked in teaching hospitals and 11.9% in private hospitals; as expected, the majority (85.3%) of gastroenterology fellows practiced in teaching hospitals. Gastroenterologists who participated to the study were similar to non‐participants in terms of gender, area of residence and hospital setting, but were significantly older (*p *= .02), whereas no difference was found between participant and non‐participant gastroenterology fellows. Table 1 shows demographic and professional characteristics of gastroenterologists and gastroenterology fellows.

**TABLE 1:** Characteristics of gastroenterologists and gastroenterology fellows

	Gastroenterologists			Gastroenterology fellows		
	Non‐participants *n *= 255	Participants *n* = 279	*p*‐Value	Non‐participants *n* = 79	Participants *n* = 61	*p*‐Value
	*n* (%)	*n* (%)		*n* (%)	*n* (%)	
Gender						
Male	169 (66.3)	176 (63.1)		41 (51.9)	36 (59)	
Female	86 (33.7)	103 (36.9)	.44	38 (48.1)	25 (41)	.40
Age group*						
<30	0	1 (0.3)		39 (49.4)	30 (49.2)	
30–40	60 (23.6)	53 (19.1)		40 (50.6)	31 (50.8)	
41–50	74 (29.1)	55 (19.8)		0	0	
51–60	70 (27.6)	103 (37.1)		0	0	
>60	50 (19.7)	66 (23.7)	.02	0	0	0.98
Geographic area°						
North‐East	54 (21.3)	51 (18.3)		15 (19)	13 (21.3)	
North‐West	53 (20.9)	62 (22.2)		9 (11.4)	11 (18)	
Center	65 (25.6)	78 (28)		26 (32.9)	20 (32.8)	
South	82 (32.3)	88 (31.5)	.80	29 (36.7)	17 (27.9)	.57
Hospital setting*						
Community hospital	158 (62.2)	175 (62.9)		2 (2.5)	6 (9.8)	
Teaching hospital	77 (30.3)	70 (25.2)		73 (92.4)	52 (85.3)	
Private hospital	19 (7.5)	33 (11.9)	.14	4 (5.1)	3 (4.9)	.18

*Missing data for one non‐participant and one participant gastroenterologist. °Missing data for one non‐participant gastroenterologist.

### 3.2 Diagnosis of *H. pylori* infection

The most preferred test for the diagnosis of *H. pylori* infection among gastroenterologists and fellows was UBT (40.4% and 57.6%, respectively), followed by stool antigen test (SAT) (32.1% and 30.5%, respectively). The majority of gastroenterologists (61.3%) and fellows (70%) would prefer UBT for the confirmation of *H. pylori* eradication.

Almost all gastroenterologists (85.3%) and fellows (88.3%) correctly prescribed a test for *H. pylori* eradication at least 4 weeks after the end of treatment.

Unfortunately, culture or molecular tests to assess antimicrobial susceptibility of *H. pylori* were available for only one third of gastroenterologists (33.7%). A significant higher proportion of fellows referred that such tests were available in their hospital (75%, *p* < .001). Table 2 shows practice patterns of gastroenterologists and gastroenterology fellows in the diagnosis of *H. pylori* infection.

**TABLE 2:** Diagnosis of *H. pylori* infection

	Gastroenterologists *n* = 279	Gastroenterology fellows *n* = 61	*p* Value
	*n* (%)	*n* (%)	
Preferred test to diagnose *H. pylori* infection			
Participants, *n*.	265	59	
^13^C‐Urea breath test	107(40.4)	34 (57.6)	
Stool antigen test	85 (32.1)	18 (30.5)	
Serology	5 (1.9)	0	
Histology	53 (20)	6 (10.2)	
Rapid urease test	15 (5.7)	1 (1.7)	.07
Preferred test to assess *H. pylori* eradication			
Participants, *n*.	271	60	
^13^C‐Urea breath test	166 (61.3)	42 (70)	
Stool antigen test	86 (31.7)	14 (23.3)	
Serology	4 (1.5)	1 (1.7)	
Histology	9 (3.3)	3 (5)	
Rapid urease test	6 (2.2)	0	.48
Interval between the end of anti‐*H. pylori* treatment and testing for eradication			
Participants, *n*.	279	60	
2 weeks	10 (3.6)	2 (3.3)	
4 weeks	141 (50.5)	33 (55)	
6 weeks	49 (17.6)	12 (20)	
8 weeks	48 (17.2)	8 (13.3)	
>8 weeks	31 (11.1)	5 (8.3)	.88
Availability of antimicrobial susceptibility *H. pylori* testing			
Participants, *n*.	267	60	
No	167 (62.5)	13 (21.7)	
Yes, both culture and genetic test	16 (6)	20 (33.3)	
Yes, only culture	74 (27.7)	25 (41.7)	
Yes, only genetic test	0	0	
I do not know	10 (3.8)	2 (3.3)	<.001

### 3.3 Treatment of *H. pylori* infection

Nearly half of gastroenterologists (45%) reported that less than 50% of their patients with *H. pylori* infection were naïve to treatment, which means that they treated more often patients with previous eradication failures. No significant difference was found with gastroenterology fellows.

About half of gastroenterologists (59%) and fellows (52.5%) reported that local prevalence of clarithromycin resistance was ≥15%, whereas for 18% of gastroenterologists and 11.9% of fellows was <15%; the prevalence of clarithromycin resistance was unknown for 22.2% of gastroenterologists and 35.6% of fellows.

Before prescribing a therapy, almost all gastroenterologists (91%) and a significant lower proportion of fellows (81.4%, *p *= .03), correctly investigated the previous use of macrolides or fluoroquinolones.

**FIGURE 1:**
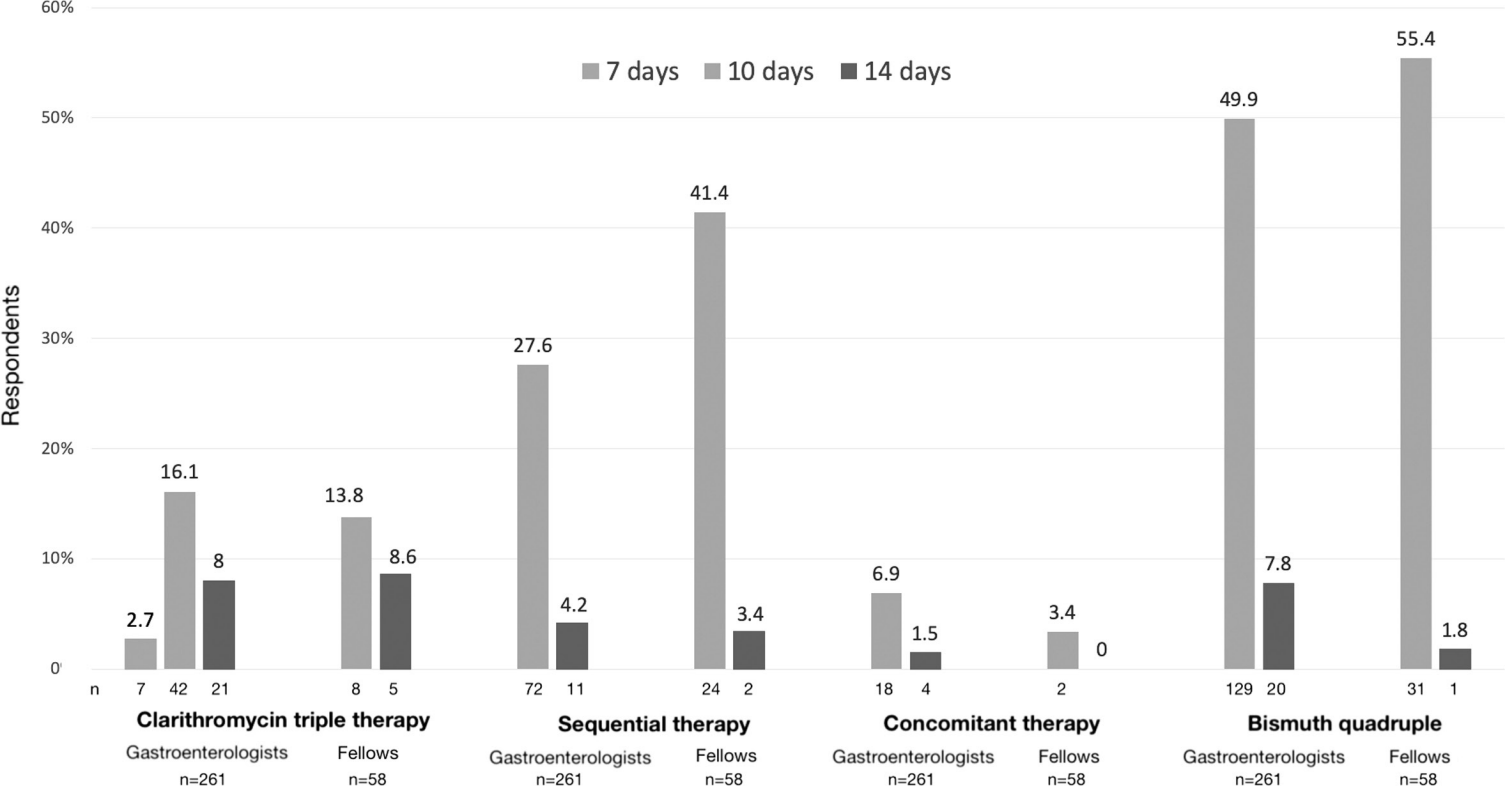
Preferred duration of first‐line treatment by gastroenterologists and gastroenterology fellows

The most preferred first‐line therapy for *H. pylori* infection among gastroenterologists and fellows was sequential therapy (31.8% and 44.8%, *p *= .58, respectively), followed by single‐capsule bismuth quadruple therapy (31% and 27.6%, *p *= .61, respectively), and clarithromycin triple therapy (26.8% and 22.4%, *p *= .49, respectively). Only a minority of gastroenterologists (8%) and fellows (3.4%) would prefer concomitant therapy. As regard the duration, the majority of gastroenterologists (82.7%, 216/261) and fellows (86.2%, 50/58) prescribed a 10‐day therapy. Figure 1 shows the duration of first‐line treatment by type of regimen. Notably, only 30% (22/70) of gastroenterologists and 38.5% (5/13) of fellows prescribed the clarithromycin triple therapy for the recommended duration of 14 days.

The most preferred second‐line regimen among gastroenterologists and fellows was single‐capsule bismuth quadruple therapy (57.8% and 57.1%, respectively), followed by levofloxacin triple therapy (31.4% and 30.4%, respectively). Again, the most preferred duration of second‐line therapy was 10 days for both gastroenterologists (85.2%, 196/230) and gastroenterology fellows (87.7%, 43/49).

After failure of second‐line therapy, the majority of gastroenterologists (72.6%) and fellows (62.5%) still preferred an empirical rather than susceptibility‐guided therapy. Either single‐capsule bismuth quadruple therapy or levofloxacin triple therapy, if not already used, was the most frequent third‐line therapy for both gastroenterologists (49.8%) and fellows (37.5%).

Only after failure of third‐line therapy, the most preferred strategy was a susceptibility‐guided therapy based on culture or molecular test; this approach was significantly more frequent among fellows than gastroenterologists (71.4% vs. 46.7%, *p *< .0001). Table 3 shows practice patterns of gastroenterologists and gastroenterology fellows in the treatment of *H. pylori* infection.

**TABLE 3:** Treatment of *H. pylori* infection

	Gastroenterologists *n *= 279	Gastroenterology fellows *n* = 61	*p* Value
	*n* (%)	*n* (%)	
Proportion of patients with *H. pylori* infection treated with first‐line therapy (naive patients)			
Participants, *n*.	269	60	
<30%	56 (20.8)	8 (13.3)	
30%–50%	65 (24.2)	11 (18.3)	
50%–70%	58 (21.6)	17 (28.3)	
>70%	87 (32.3)	22 (36.7)	
I do not know	3 (1.1)	2 (3.3)	.28
Regional prevalence of *H. pylori* clarithromycin resistance			
Participants, *n*.	266	59	
<15%	50 (18.0)	7 (11.9)	
≥15%	157 (59)	31 (52.5)	
I do not know	59 (22.2)	21 (35.6)	.07
Investigation on previous use of macrolides and fluoroquinolones.			
Participants, *n*.	268	59	
No	24 (9)	11 (18.6)	
Yes, for both macrolides and quinolones	218 (81.3)	41 (69.5)	
Yes, but only for macrolides	24 (8.9)	4 (6.8)	
Yes, but only for fluoroquinolones	2 (0.8)	3 (5.1)	.01
Preferred first‐line therapy for *H. pylori* infection			
Participants, *n*.	261	58	
Clarithromycin‐based triple therapy	70 (26.8)	13 (22.4)	
Sequential therapy	83 (31.8)	26 (44.8)	
Single‐capsule bismuth quadruple therapy	81 (31)	16 (27.6)	
Concomitant therapy	22 (8.4)	2 (3.4)	
Hybrid therapy	2 (0.8)	0	
Other	3 (1.1)	1 (1.7)	.41
Preferred second‐line therapy for *H. pylori* infection			
Participants, *n*.	258	56	
Repeat the same treatment, possibly for more days	1 (0.4)	2 (3.6)	
Sequential or concomitant therapy	11 (4.3)	1 (1.8)	
Single‐capsule bismuth quadruple therapy	149 (57.8)	32 (57.1)	
Levofloxacin‐based triple therapy	81 (31.4)	17 (30.4)	
Other	16 (6.2)	3 (7.2)	.22
Preferred third‐line therapy for *H. pylori* infection			
Participants, *n*.	259	56	
Repeat the same second‐line treatment, possibly for more days	4 (1.5)	2 (3.6)	
Single‐capsule bismuth quadruple therapy or levofloxacin‐based triple therapy	129 (49.8)	21 (37.5)	
Rifabutin‐based triple therapy	18 (6.9)	3 (5.4)	
Susceptibility‐guided therapy based on culture or genetic test	71 (27.4)	21 (37.5)	
Other	37 (14.3)	9 (16.1)	.36
Management of patient after failure of three lines of treatment			
Participants, *n*.	259	56	
No further eradication therapy for *H. pylori*	44 (17)	1 (1.8)	
Rifabutin‐based triple therapy	44 (17)	5 (8.9)	
Susceptibility‐guided therapy based on culture or genetic test	121 (46.7)	40 (71.4)	
Referral the patient to a colleague with more experience in *H. pylori* treatment	24 (9.3)	7 (12.5)	
Other	26 (10)	3 (5.4)	<.001

### 3.4 Management of *H. pylori* according to the hospital setting

Compared with community hospitals, a significant higher proportion of physicians in teaching hospitals used UBT for confirmation of *H. pylori* eradication (69.8% vs. 56.3%, *p *= .02). Culture and genetic tests to assess *H. pylori* susceptibility were more frequently available in teaching than community hospitals (61.2% vs. 33.1%, respectively, *p* < .00001). This would partially explain the previous finding that antimicrobial susceptibility tests were more available for fellows than gastroenterologists, as fellows practiced in teaching hospitals more than gastroenterologists (85.3% vs. 25.2%, respectively *p* < .0001) (Table 4).

**TABLE 4:** Diagnosis of *H. pylori* infection in community and teaching hospitals

	Community hospital *n* = 181	Teaching hospital *n* = 122	*p* Value
	n (%)	*n* (%)	
Preferred test to diagnose *H. pylori* infection			
Participants, *n*.	173	116	
^13^C‐Urea breath test	69 (39.9)	52 (44.8)	
Stool antigen test	62 (35.8)	36 (31)	
Serology	3 (1.7)	2 (1.7)	
Histology	32 (18.5)	17 (14.7)	
Rapid urease test	7 (4.1)	9 (7.8)	.52
Preferred test to assess *H. pylori* eradication			
Participants, *n*.	176	119	
^13^C‐Urea breath test	99 (56.3)	83 (69.8)	
Stool antigen test	62 (35.2)	32 (26.9)	
Serology	2 (1.1)	2 (1.7)	
Histology	10 (5.7)	1 (0.8)	
Rapid urease test	3 (1.7)	1 (0.8)	.07
Interval between end of anti‐*H. pylori* treatment and testing for eradication			
Participants, *n*.	181	121	
2 weeks	6 (3.3)	3 (2.5)	
4 weeks	82 (45.3)	77 (63.6)	
6 weeks	37 (20.4)	18 (14.9)	
8 weeks	40 (22.1)	12 (9.9)	
>8 weeks	16 (8.8)	11 (9.1)	.01
Availability of antimicrobial susceptibility *H*. *pylori* testing			
Participants, *n*.	175	116	
No	110 (62.9)	41 (35.3)	
Yes, both culture and genetic test	7 (4)	27 (23.3)	
Yes, only culture	51 (29.1)	44 (37.9)	
Yes, only genetic test	0	0	
I do not know	7 (4)	4 (3.5)	<.001

Note

Community hospitals: *n*.175 gastroenterologists and 6 gastroenterology fellows. Teaching hospitals: *n*. 70 gastroenterologists and 52 gastroenterology fellows.

There were no significant differences between teaching and community hospitals for the treatment of *H. pylori* infection, apart from a higher proportion of physicians in teaching hospitals who preferred a concomitant therapy at first line (10.5% vs. 3.5%, respectively, *p *= .03). After failure of three lines of treatment, more physicians in teaching than community hospitals preferred a susceptibility‐guided therapy (63.4% vs. 45.2%, respectively, *p *= .003) (Table 5).

**TABLE 5:** Treatment of *H. pylori* infection in community and teaching hospitals

	Community hospital *n* = 181	Teaching hospital *n* = 122	*p* Value
	*n* (%)	*n* (%)	
Proportion of patients with *H. pylori* infection treated with first‐line therapy (naïve patients)			
Participants, *n*.	175	118	
<30%	30 (17.1)	28 (23.7)	
30%–50%	44 (25.1)	21 (17.8)	
50%–70%	41 (23.4)	29 (24.6)	
>70%	58 (33.1)	37 (31.4)	
I do not know	2 (1.1)	3 (2.5)	.38
Regional prevalence of clarithromycin resistance			
Participants, *n*.	175	114	
<15%	32 (18.3)	19 (16.7)	
≥15%	107 (61.1)	61 (53.5)	
I do not know	36 (20.6)	34 (29.8)	.20
Investigation on previous use of macrolides and quinolones before therapy			
Participants, *n*.	173	117	
No	14 (8.1)	17 (14.5)	
Yes, for both macrolides and quinolones	142 (82.1)	88 (75.2)	
Yes, but only for macrolides	16 (9.3)	11 (9.4)	
Yes, but only for fluoroquinolones	1 (0.6)	1 (0.9)	.36
Preferred first‐line therapy for *H. pylori* infection			
Participants, *n*.	171	113	
Clarithromycin‐based triple therapy	43 (25.2)	34 (30.1)	
Sequential therapy	53 (31)	43 (38.1)	
Single‐capsule bismuth quadruple therapy	54 (31.6)	30 (26.6)	
Concomitant therapy	18 (10.5)	4 (3.5)	
Hybrid therapy	1 (0.6)	1 (0.8)	
Other	2 (1.2)	1 (0.8)	.17
Preferred second‐line therapy for *H. pylori* infection			
Participants, *n*.	168	109	
Repeat the same treatment, possibly for more days	1 (0.6)	2 (1.8)	
Sequential or concomitant therapy	9 (5.4)	1 (0.9)	
Single‐capsule bismuth quadruple therapy	98 (58.3)	61 (55.9)	
Levofloxacin‐based triple therapy	50 (29.8)	38 (34.9)	
Other	10 (6)	7 (6.4)	.29
Preferred third‐line therapy for *H. pylori* infection			
Participants, *n*.	168	110	
Repeat the same second‐line treatment, possibly for more days	3 (1.8)	3 (2.7)	
Single‐capsule bismuth quadruple therapy Or levofloxacin‐based triple therapy	80 (47.6)	52 (47.2)	
Rifabutin‐based triple therapy	7 (4.2)	7 (6.4)	
Susceptibility‐guided therapy based on culture or genetic test	51 (30.4)	34 (30.9)	
Other	27 (16.1)	14 (12.7)	.84
Management of patient after failure of three lines of treatment			
Participants, *n*.	166	112	
No further eradication therapy for *H. pylori*	30 (18.1)	8 (7.1)	
Rifabutin‐based triple therapy	27 (16.3)	13 (11.6)	
Susceptibility‐guided therapy based on culture or genetic test	75 (45.2)	71 (63.4)	
Referral to a gastroenterologist with more experience in *H. pylori* treatment	17 (10.2)	11 (9.8)	
Other	17 (10.2)	9 (8)	.02

Note

Community hospitals: *n*.175 gastroenterologists and 6 gastroenterology fellows. Teaching hospitals: *n*. 70 gastroenterologists and 52 gastroenterology fellows.

## 4 DISCUSSION

This study describes practice patterns of gastroenterologists and gastroenterology fellows in the diagnosis and treatment of *H. pylori* infection in Italy and their adherence to international guidelines.^9^, ^10^, ^11^

In accordance with Maastricht V/Florence Consensus Report,^9^ UBT was the most preferred method for both diagnosis of *H. pylori* infection and confirmation of eradication. These data are in line with previous studies reporting that UBT was the most common method for the pre‐ and post‐treatment diagnosis of *H. pylori* infection among gastroenterologists in Europe and Asia; the UBT was used for confirmation of eradication in 73% and 88% of cases by European^14^ and Chinese^15^ gastroenterologists, respectively. It is well known that antibiotics should be discontinued at least 4 weeks before testing in order to avoid false‐negative test results^9^; in our study, almost all gastroenterologists and trainees properly performed the test at least 4 weeks after the end of therapy. This is in contrast with Chinese survey showing that only 75% of clinicians assessed accurately the effect of treatment performing the test at least 4 weeks after the completion of therapy.^15^

We found that the culture or molecular tests to assess antimicrobial susceptibility of *H. pylori* are not widely available in Italy. In fact, such tests were available for only one third of the gastroenterologists, a rate that reached 61% in teaching hospitals. Antimicrobial susceptibility testing is not available in most centers in North America,^11^ and this is likely to happen also in Europe. In the future, molecular tests applied to fecal samples, if proven reliable, can help improving the assessment of antibiotic resistance of *H. pylori*, thus obviating the need for endoscopy.^21^ Indeed, a susceptibility‐guided first‐line therapy could improve the efficacy of eradication regimen, decrease indirect costs related to treatment failure, and counteract the increasing emergence of antimicrobial‐resistant *H. pylori* strains.^22^, ^23^, ^24^

There is evidence that a previous course of clarithromycin and quinolone is associated with an increased risk of antibiotic resistance of *H. pylori* to that antimicrobial agent,^25^ that will consequently impact on the outcome of eradication treatment.^26^ Thus, guidelines recommend to investigate the previous use of antibiotics in order to derive an individual‐based information on likely antimicrobial resistance of *H. pylori*.^9^ This approach may be useful for the choice of the best therapy, in particular in areas of low or unknown clarithromycin resistance. Accordingly, we found that almost all gastroenterologists and fellows investigated a previous use of macrolides or quinolones before prescribing an eradication therapy.

Current guidelines advocate that the choice of the first‐line *H. pylori* eradication therapy should be based on the knowledge of the regional prevalence of clarithromycin antibiotic resistance.^9^, ^10^, ^11^ For about 60% of gastroenterologists, the regional prevalence of clarithromycin resistance was >15%, whereas for about 20% of them was <15% and for the remaining 20% was unknown. Unfortunately, there are no epidemiological studies on representative sample of patients that assessed the prevalence of clarithromycin resistance in Italy. Some studies carried out in a few clinical centers enrolling selected samples of patients reported a high prevalence of clarithromycin resistance, around 30%.^27^, ^28^ On the other hand, a meta‐analysis, including seven Italian studies showed a pooled prevalence of clarithromycin resistance of 15% with a lower limit of the 95% confidence interval of 11%.^7^ The European registry of *H. pylori* management reported a clarithromycin resistance of 11.9% in the Center of Europe, a geographic area including only Italy and France.^14^ Indeed, the real prevalence of clarithromycin resistance remains still uncertain and may vary across regions in Italy.

Sequential therapy was the most preferred first‐line treatment for *H. pylori* infection by gastroenterologists and gastroenterology fellows in Italy. These data seem to be true: the European registry reported that sequential therapy accounted for 61% of first‐line therapies in Centre Europe, where about 90% of prescriptions come from Italy.^14^ Sequential therapy, which is a 5‐day amoxicillin‐containing double therapy followed by a 5‐day clarithromycin triple therapy, was initially designed to overcome the issue of clarithromycin resistance. Unfortunately, sequential regimen is undermined by single and, especially, dual resistance to clarithromycin and metronidazole.^29^, ^30^ Eradication rates with sequential therapy are consistently lower than that of concomitant o bismuth quadruple therapy.^14^, ^31^, ^32^ Based on these data, all international guidelines have discouraged the use of sequential therapy in clinical practice.^9^, ^10^, ^11^ Indeed, sequential therapy has been falling into disuse in Europe accounting for only about 8% of first‐line treatments; this regimen provided eradication rates <90% across all European countries, including Italy.^14^ However, several reasons may explain the current popularity of this un‐recommended regimen in Italy. Sequential therapy was developed in Italy in the year 2000 and was proposed as one of the first‐line therapies by national guidelines in 2015,^33^ before the publication of the updated international recommendations. In addition, some Italian studies reported an unexpected, good performance of this regimen with eradication rates >90%, even in patients with clarithromycin resistant strains.^34^, ^35^

In our study, about 80% of gastroenterologists referred that the prevalence of clarithromycin resistance in their region was high or unknown, but only 40% would prefer bismuth quadruple or concomitant therapies for the first‐line treatment of *H. pylori* infection. This means that at least half of gastroenterologists prescribed a non‐recommended regimen in naïve patients. Bismuth quadruple therapy was preferred by only one third of gastroenterologists and trainees in gastroenterology; this finding would confirm that the use of bismuth quadruple therapy at first line is still uncommon in Europe; however, a time‐trend analysis showed an increase in the use of this regimen from 0.2% of prescriptions in 2013 to 22% in 2018 in Europe.^14^ Bismuth quadruple therapy was the most preferred option for the first‐line treatment of *H. pylori* infection in China, but again this regimen was used only by 57% of gastroenterologists.^15^

Only a minority of gastroenterologists and gastroenterology fellows preferred clarithromycin triple therapy for the first‐line treatment of *H. pylori*. The use of clarithromycin triple therapy by gastroenterologists has declined over time in Europe, going from >50% of prescription in 2013 to 35% in 2018.^14^ Notably, we found that only about one third of participants who preferred a clarithromycin triple therapy prescribed a 14‐day regimen. A Cochrane meta‐analysis showed that the optimal duration of triple therapy is 14 days, which is now the recommended treatment duration of clarithromycin triple therapy.^36^ Unfortunately, the use of triple therapy for less than 14 days is still common among gastroenterologists in the eradication of *H. pylori*.^36^, ^37^

Single‐capsule bismuth quadruple therapy was the most preferred second‐line therapy by gastroenterologists, followed by levofloxacin triple therapy, which is in agreement with international recommendations.^9^, ^10^, ^11^ After failure of a second‐line treatment, guidelines suggest a therapy guided by antimicrobial susceptibility testing or, in alternative, if such tests are not available, an empirical therapy with a regimen that had not been already used.^9^, ^10^, ^11^ In our study, the majority of gastroenterologists and trainees would prefer an empirical therapy, in particular single‐capsule bismuth quadruple therapy or levofloxacin triple therapy, and this would reflect the scarce availability of culture or molecular tests in clinical practice. Only after failure of third‐line therapy, the most frequent approach of gastroenterologists was a therapy driven by antimicrobial susceptibility testing; this approach was significantly more frequent among fellows than gastroenterologists for the greater availability of susceptibility testing in teaching than community hospitals.

To our knowledge, this is the most comprehensive study assessing practice patterns of gastroenterologists and gastroenterology fellows in the diagnosis and treatment of *H. pylori* infection in Europe. Previous studies reported either attitudes of primary care physicians^18^ or practices of gastroenterologists, but not gastroenterology fellows, with particular focus on the first‐line treatment of *H. pylori*.^14^ Another comprehensive survey on the adherence of gastroenterologists to guideline for the management of *H. pylori* infection was carried out in China.^15^ In addition, this is the first study providing data on the availability of culture and molecular tests for antimicrobial susceptibility of *H. pylori* in clinical practice in Europe.

This study has several limitations. The main limitation is the low participation rate of about 50% for gastroenterologists and 40% for gastroenterology fellows. However, the participation rate was high compared to that of other surveys on the same topic, ranging from 11% to 30%.^18^, ^20^, ^38^ We think that our study sample is not too far to be representative of gastroenterologists and gastroenterology fellows in Italy. The National Congress of Digestive Diseases ‐ FISMAD is the annual Congress of the three major scientific societies of digestive diseases, thus gastroenterologists and trainees who attend this Congress are very likely to represent the entire population of gastroenterologists and gastroenterology fellows in Italy. In addition, the characteristics of participants were similar to that of non‐participants, apart from age, thus minimizing the introduction of selection bias. Other limitations of this study are those inherent to questionnaire‐based surveys, such as about telling the truth, with responses that may be skewed toward adherence to guidelines. Finally, there is a general delay from publication of recommendations to their implementation in routine clinical practice,^39^ and our survey was carried out only after 6–12 months since the publication of the guidelines.

In conclusion, the management of *H. pylori* infection by gastroenterologists and gastroenterology fellows is in line with guidelines’ recommendations in Italy, apart for the first‐line treatment of *H. pylori* infection. In contrast with international recommendations, sequential therapy is the most preferred first‐line therapy, whereas bismuth and non‐bismuth quadruple therapies are still underused. A minority of gastroenterologists and fellows would prefer clarithromycin triple therapy, but only one third uses the recommended 14‐day regimen. Unfortunately, this is a cause of high rate of eradication failures and may negatively affect the practice of primary care physicians in the treatment of *H. pylori*. Finally, antimicrobial susceptibility tests are not widely available in clinical practice; thus, physicians would prefer a susceptibility‐guided therapy only after failure of three lines of treatment. In future, scientific societies should implement targeted educational interventions in order to improve the adherence of gastroenterologists and gastroenterology fellows to guidelines’ recommendations for the first‐line treatment of *H. pylori* infection.

## CONFLICT OF INTEREST

The authors have no conflict of interest to declare.

## AUTHOR CONTRIBUTIONS

RMZ and FB conceived the study and drafted the protocol. RMZ, MR, and LF performed statistical analysis and drafted the manuscript. All the other authors revised the manuscript and approved the final version.

## Supporting information

Appendix S1
