# Supplementary material for: Adherence to international guidelines for the management of Helicobacter pylori infection among gastroenterologists and gastroenterology fellows in Italy: A Survey of the Italian Federation of Digestive Diseases ‐ FISMAD
Source: Helicobacter. 2021 Nov 11;27(1):e12862. doi: 10.1111/hel.12862 (PMC9286052; doi:10.1111/hel.12862)
Supplement: Supplementary file 1 — Appendix S1 [file HEL-27-0-s001.docx]

**Supplementary Appendix 1: Questionnaire**

**SECTION I: DEMOGRAPHICS AND PROFESSIONAL CHARACTERISITCS**

**1. Gender**

 Male

 Female

1. **Age (years)**

 < 30

 30 - 40

 41 - 50

 51 - 60

 > 60

1. **Macro-area of residence**

 Nord-West **(**Valle d’Aosta, Piedmont, Lombardy, Liguria)

 Nord-East **(**Friuli-Venezia Giulia, Veneto, Emilia Romagna, Trento/ Bolzano)

 Center **(**Tuscany, Marche, Umbria, Lazio)

 South and Islands **(**Abruzzo, Molise, Campania, Apulia, Basilicata, Calabria, Sicily,

Sardinia)

1. **In which hospital do you work?**

 Community Hospital

 Teaching Hospital

 Private Hospital

1. **Are you a Gastroenterologist or a Gastroenterology fellow ?**

 Gastroenterologist

 Gastroenterology fellow

**SECTION II:** **DIAGNOSIS**

1. **Which test do you prefer to diagnose *H. pylori* infection ?**

^13^C-Urea breath test

 Stool antigen test

 Serology

 Histology

 Rapid urease test

1. **Which test do you prefer to assess *H. pylori* eradication?**

^13^C-Urea breath test

 Stool antigen test

 Serology

 Histology

 Rapid urease test

1. **How many weeks after the end of *H. pylori* treatment do you test for eradication?**

 2 weeks

 4 weeks

 6 weeks

 8 weeks

 > 8 weeks

4. **Are** **culture or genetic tests to assess *H. pylori* antimicrobial susceptibility available**

**in your hospital ?**

 No

 Yes, both culture and genetic tests

 Yes, only culture

 Yes, only genetic test

 I do not know

**SECTION III:** **TREATMENT**

1. **What is the proportion of your patients with *H. pylori* infection naïve to treatment ?**

 < 30%

 30% - 50%

 50% - 70%

 > 70%

 I do not know

1. **What is in your region the prevalence of clarithromycin resistance of *H. pylori* ?**

 < 15 %

 ≥15 %

 I do not know

1. **Before a treatment for *H. pylori* infection, do you investigate a previous use of macrolides (clarithromycin/azithromycin) or quinolones (levofloxacin/ciprofloxacin) ?**

 No

 Yes, I investigate for both macrolides and quinolones

 Yes, but only for macrolides

 Yes, but only for quinolones

1. **What is your preferred therapy for the first-line treatment of *H. pylori* infection ?**

 7-day clarithromycin triple therapy

 10-day clarithromycin triple therapy

 14-day clarithromycin triple therapy

 10-day sequential therapy

 14-day sequential therapy

 10-day non-bismuth concomitant quadruple therapy

 14-day non-bismuth concomitant quadruple therapy

 10-day single-capsule bismuth quadruple therapy

 14-day single-capsule bismuth quadruple therapy

 10-day hybrid therapy

 14-day hybrid therapy

 Other

1. **What is your preferred therapy for the second-line treatment of *H. pylori* infection ?**

 Repeat the first-line treatment, possibly for more days

 Sequential or concomitant therapy

 10-day levofloxacin triple therapy

14-day levofloxacin triple therapy

 10-day single-capsule bismuth quadruple therapy

14-day single-capsule bismuth quadruple therapy

 Other

1. **What is your preferred therapy for the third-line treatment of *H. pylori* infection?**

 Repeat the second-line treatment, possibly for more days

 Single-capsule bismuth quadruple therapy or levofloxacin triple therapy, if not already

used

 Rifabutin-based triple therapy

 Susceptibility-guided therapy based on culture or genetic test

 Other

1. **How do you manage *H. pylori* infection after failure of three lines of treatment ?**

 I no longer perform any eradication therapy

 I prescribe a rifabutin-based triple therapy, if not already used

 I prescribe a susceptibility-guided therapy based on culture or genetic test, if not already

done

 I refer the patient to a colleague with more experience in *H. pylori* treatment

 Other
